# Metabolic regulators of enigmatic inflammasomes in autoimmune diseases and crosstalk with innate immune receptors

**DOI:** 10.1111/imm.13326

**Published:** 2021-05-02

**Authors:** Gisela Jimenez‐Duran, Martha Triantafilou

**Affiliations:** ^1^ Immunology Network GlaxoSmithKline Stevenage UK; ^2^ Institute of Infection and Immunity School of Medicine University Hospital of Wales Cardiff University Cardiff UK

**Keywords:** complement system, GPCR signalling, gut microbiota, immunometabolic diseases, inflammasome, metabolic reprogramming

## Abstract

Nucleotide‐binding domain and leucine‐rich repeat receptor (NLR)‐mediated inflammasome activation is important in host response to microbes, danger‐associated molecular patterns (DAMPs) and metabolic disease. Some NLRs have been shown to interact with distinct cell metabolic pathways and cause negative regulation, tumorigenesis and autoimmune disorders, interacting with multiple innate immune receptors to modulate disease. NLR activation is therefore crucial in host response and in the regulation of metabolic pathways that can trigger a wide range of immunometabolic diseases or syndromes. However, the exact mode by which some of the less well‐studied NLR inflammasomes are activated, interact with other metabolites and immune receptors, and the role they play in the progression of metabolic diseases is still not fully elucidated. In this study, we review up‐to‐date evidence regarding NLR function in metabolic pathways and the interplay with other immune receptors involved in GPCR signalling, gut microbiota and the complement system, in order to gain a better understanding of its link to disease processes.

## Graphical abstract

In this study we review the current evidence with respect to inflammasome function in metabolic pathways and the interplay with other immune receptors involved in GPCR signalling, gut microbiota and the complement system, in order to gain a better understanding of its link to disease processes.

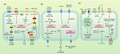

**Abbreviations**
AMP: Adenosine monophosphate
ATP: Adenosine triphosphate
CNS: Central nervous system
GSDMD: Gasdermin D
GPCR: G protein‐coupled receptor
GLUT1: Glucose transporter 1
LAT1: L‐type amino acid transporter 1
LPS: Lipopolysaccharide
mTOR: Mechanistic target of rapamycin
mtDNA: Mitochondrial DNA
NLR: Nucleotide‐binding domain and leucine‐rich repeat receptor
NPC: intracellular cholesterol transporter 1
ω‐3 FA: Omega‐3 fatty acid
OXPHOS: Oxidative phosphorylation
PI3K: Phosphoinositide 3‐kinase
PKA: Protein kinase A
PRRs: Pattern recognition receptors
ROS: Reactive oxygen species
TCA cycle: Tricarboxylic acid cycle
T1D: Type 1 diabetes

## CROSSTALK OF CELL METABOLISM AND THE NLR INFLAMMASOME

Organisms have developed host defence mechanisms that protect from microbial pathogens. The host's immune response can also regulate metabolic responses including changes in glycolysis, oxidative phosphorylation and cholesterol metabolism [1, 2, 3]. These critical biochemical reactions play a major role in immune cell function and are vital for cell maintenance, energy, proliferation and signalling [4]. Thus, it is not surprising that these metabolic processes are regulated and controlled by immune receptors, and dysregulation at any of these pathways can have an impact on organismal health and therefore cause a number of different autoimmune diseases.

**FIGURE 1:**
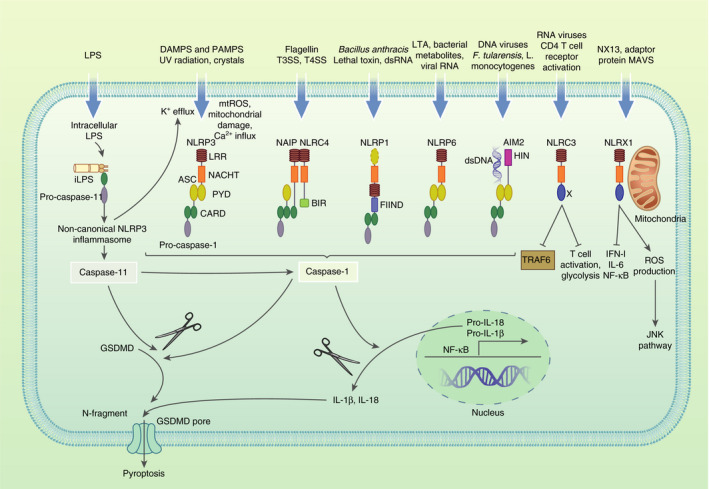
**Activation of NLRs, non‐canonical and canonical inflammasomes.** Structure and activating ligands of canonical NLRP3, NLRC4, NLRP1 (human NLRP1 inflammasome contains PYD, mouse NLRP1 does not), NLRP6 and AIM2 inflammasomes, as well as non‐canonical NLRP3 inflammasome, and NLRC3 and NLRX1, which are non‐inflammasome forming NLRs. Human and mouse NLRs and AIM2 get activated in response to distinct ligands. Double‐stranded RNA (dsRNA) is an activator of human NLRP1 while anthrax lethal toxin activates murine NLRP1B. NLRC4 inflammasome gets activated by T3SS/T4SS indirectly by detecting flagellin, or directly by detecting the T3SS rod protein. Mouse NAIP5 and 6 detect flagellin, mouse NAIP 1 and 2 as well as human NAIP respond to proteins of type III secretion systems (T3SS). Upon ligand binding, NAIP and NLRC4 assemble into an oligomerized inflammasome. Among several bacterial ligands of AIM2, *Francisella tularensis* and *Listeria monocytogenes* act as activators of mouse AIM2 inflammasome. DNA and RNA viruses are ligands to mostly human AIM2 inflammasome. Canonical activation of inflammasomes results in oligomerization of ASC into speck complexes, recruiting pro‐caspase 1 leading to self‐cleavage and activation of caspase 1. Caspase 1 cleaves pro‐IL‐1β (produced by NFκB‐induced upregulation) and pro‐IL‐18, as well as GSDMD cleavage. The N‐terminal fragment of GSDMD forms pores on the cell membrane and enables secretion of IL‐1β and IL‐18, with subsequent pyroptosis. Non‐canonical NLRP3 inflammasome activation occurs via human caspase 4 and 5 or murine caspase 11. Sensing of intracellular LPS by pro‐caspase 11 leads to caspase 11 activation and subsequent GSDMD cleavage, as well as K^+^ efflux promoting the activation of NLRP3‐ASC‐caspase 1 pathway

Inflammasomes are an essential part of host defence and have been also implicated in metabolic regulation [5, 6]. Inflammasomes are multiprotein complexes that become activated in response to a variety of physiological and pathogenic stimuli. Canonical inflammasome assembly through caspase 1 activation is highly regulated and two different danger signals are required for its activation, priming and activation. Priming is initiated mainly by activation of PRRs, including TLR4 and cytokine receptors, leading to nuclear translocation of nuclear factor κB (NF‐κB) and subsequent upregulation and translation of pro‐interleukin‐1β (pro‐IL‐1β) (pro‐IL‐18 is constitutively expressed). Inflammasome assembly and activation require cytosolic sensing of pathogen‐associated molecular patterns (PAMPS) or danger‐associated molecular patterns (DAMPS) by a nucleotide‐binding domain and leucine‐rich repeat receptor (NLR) or absent in melanoma 2 (AIM2)‐like receptors (ALR). NLRs and ALRs engage caspase 1, in most cases requiring the recruitment and binding of the ASC (apoptotic‐speck‐containing protein with a CARD), to catalyse proteolytic cleavage of pro‐IL‐1β and pro‐IL‐18, as well as gasdermin D (GSDMD) cleavage, forming pores on the cell membrane leading to pyroptosis (Figure 1) [7, 8]. The main NLRs that have been linked with metabolic regulation include pyrin domain‐containing protein 1 (NLRP1), NLRP3, NLRP6, NLRP12, NLR family CARD (caspase activation and recruitment) domain‐containing 4 (NLRC4), NAIP (neuronal apoptosis inhibitor protein), NLRC3 and NLRX1, and members of the PYHIN family include absence in melanoma 2 (AIM2) [6, 9].

## METABOLIC REGULATION AND INFLAMMASOME INTERACTIONS

### NLRP3

Activation of the NLRP3 inflammasome can be modulated by the metabolic state of a cell and acts as a general sensor of cellular homeostasis. For instance, increased AMP leads to inhibition of inflammasome activation by activating the nutrient sensor AMP‐dependent protein kinase (AMPK), which causes a metabolic switch from glycolysis (and energy‐consuming pathways linked to high cellular activity) to OXPHOS, which is linked to anti‐inflammatory, quiescent and contracting cell responses [10]. In addition, glycolytic and Krebs cycle enzymes have been reported to induce NLRP3 inflammasome activation. The glycolytic enzyme pyruvate kinase M2 (PKM2) activates the NLRP3 inflammasome in macrophages stimulated with LPS via hypoxia‐inducible factor 1‐alpha (HIF1α) regulation, causing maintained IL‐1β production [11]. Other signals linked to cellular stress that activate NLRP3 include ATP influx, reactive oxygen species (ROS) and increased concentration of intracellular calcium ([Ca^2+^]i) (Figure 1) as a result of ion fluxes or changes in glucose and lipid metabolism [12]. Recent studies showing NLRP3 activation by changes in lipid metabolism, such as increased endoplasmic reticulum cholesterol levels via NPC1, or saturated fatty acids crystallization via lysosomal destabilization, are crucial to provide further key roles for inflammasomes in lipid metabolism‐related diseases [13]. Metabolic regulation of the NLRP3 inflammasome has been extensively investigated and is deeply described in the following reviews [9, 14], the focus here is on the less well‐studied inflammasomes.

### NLRC4

Regulation of the NLRC4 inflammasome has been mainly linked to lipid metabolism and acts as a contributor to pathology in several immunometabolic diseases. Murine NAIP5 (NLR family, apoptosis inhibitory protein 5)/NLRC4 inflammasome activation by cytosolic delivery of bacterial flagellin has been shown to cause an eicosanoid storm, causing an uncontrolled release of signalling lipids such as prostaglandins, leading to inflammation, vascular fluid loss and death in mice. This process was dependent on the initial caspase 1 activation by NAIP5/NLRC4, triggering Ca^2+^ influx and cyclooxygenase 1 stimulation, a crucial enzyme in lipid metabolism responsible for prostaglandin biosynthesis, leading to the eicosanoid storm [15].

Importantly, NLRC4 inflammasome activation has been involved in several metabolic diseases such as colitis‐associated tumorigenesis [16], obesity‐associated breast cancer progression, non‐alcoholic steatohepatitis (NASH), diabetic nephropathy (DN) and type 1 diabetes (T1D). Activated NLRC4 leading to IL‐1β signalling has been reported to contribute to pathology and angiogenesis in obese mice with breast cancer via adipocyte‐mediated vascular endothelial growth factor A (VEGFA) expression [17]. In addition, the omega‐3 fatty acid (ω‐3 FA) docosahexaenoic acid (DHA), which has a protective effect against NASH, has recently been shown to alleviate the palmitate (PA)‐induced inflammatory agents and lipid accumulation via suppression of human NLRC4 expression and inhibition of caspase 1 and IL‐1β cleavage. NLRC4 knockdown inhibited PA‐induced inflammation and lipid accumulation, therefore presenting DHA as a potential antagonist for NLRC4 to provide beneficial hepatoprotective effects in NASH [18].

NLRC4‐driven IL‐1β production has also been shown to contribute to pathology in DN, where NLRC4‐deficient mice showed decreased blood glucose and albumin excretion, together with preserved renal histology and lower DN progression [19]. In addition, two recent studies identified certain human NLRC4 polymorphisms associated with markers of glucose and lipid metabolism: insulin levels and higher triglyceride levels associated with NLRC4 polymorphisms from a healthy southern Brazil population [20], and positive rate of glutamic acid decarboxylase antibody (GADA) and the onset age associated with rs385076 NLRC4 polymorphism in patients with T1D in a Chinese Han population [21], indicating a clear association between NLRC4 and clinical characteristics of T1D. Further investigation should focus in the metabolic modulators involved in NLRC4 inflammasome contribution to ND and possibly T1D. In addition, it would be interesting to determine whether similar molecular mechanisms of NLRC4 leading to eicosanoid storm and inflammation that were observed in mice, such as cyclooxygenase 1 stimulation, are also involved in NASH (Table 1).

**TABLE 1:** Summary of upstream and downstream metabolic regulators interacting with NLRC4, NLRP1, AIM2, caspase 11 inflammasomes, NRLC3 and NLRX1, linked with autoimmune pathologies

Inflammasome/NLR	Effector and function	Mechanism	Pathology	References
NLRC4	**DHA.** Most present ω‐3 FA in the brain and retina, component of membrane glycerophospholipids, reduces circulating triglyceride levels.	DHA ⤍ NLRC4 suppression ⤍ decreased palmitate‐induced inflammation and lipid accumulation	Protective effect of DHA against non‐alcoholic steatohepatitis (NASH)	[18]
	**Cyclooxygenase 1**. Enzyme responsible for prostaglandin biosynthesis.	NAIP5/NLRC4 activation ⤍ Ca^2+^ influx ⤍ cyclooxygenase 1 stimulation ⤍ leading to an eicosanoid storm	Eicosanoid storm leading to inflammation, vascular fluid loss and death in mice	[15]
	**Obesity/VEGFA**. VEGFA contributes to angiogenesis and tumour progression and induces proliferation and migration of vascular endothelial cells.	NLRC4 activation IL‐1β signalling ⤍ VEGFA expression ⤍ contributing to pathology	Obesity‐associated breast cancer progression	[17]
NLRP1	**MCFA and SCFA**. Substrates of energy metabolism mostly inhibit glycolysis and stimulate lipogenesis/gluconeogenesis.	Changes in MCFA and SCFA metabolism ⤍ dysbiosis in NLRP1‐deficient mice	IBD progression	[28]
	**High glucose and glycated albumin**. Diabetes metabolites.	High glucose and glycated albumin ⤍ increased NLRP1 expression	Protective effect against diabetic kidney disease (DKD) in T1D patients.	[26]
	**High‐fat diet (HFD)**	HFD ⤍ active NLRP1, IL‐18 production ⤍ lipolysis (in mice with an activating mutation in NLRP1)	Prevention of obesity and metabolic syndrome	[34]
	**ER stress**. Engages the unfolded protein response (UPR). UPR signal activators include PERK/IRE1α.	ER stress ⤍ PERK/IRE1α ⤍ ATF4 ⤍ NLRP1 upregulation	Contribution to PERK/ATF4‐associated ISR and Chondrodysplasia.	[32, 113]
	**Streptozotocin (STZ)‐induced diabetes.** Toxic compound to pancreatic β‐cells, causing hyperglycaemia and hypoinsulinaemia.	Active NLRP1 ⤍ increased blood glucose levels ⤍ VEGF expression ⤍ pro‐inflammatory cytokine release	Contribution to pathology of diabetic retinopathy	[27]
AIM2	**PKM2**. Activated tetrameric PKM2 catalyses dephosphorylation of phosphoenolpyruvate (PEP) to pyruvate.	PKM2 ⤍ mediated glycolysis ⤍ PDK1‐EIF2AK2 ⤍ AIM2 activation, IL‐1β/IL‐18 ⤍ septic death in mice	Contribution to sepsis progression	[36]
	**Cholesterol‐25‐hydroxylase (Ch25h)‐induced 25‐HC**. Ch25h produces 25‐HC from cholesterol. 25‐HC has antiviral activity.	Type I IFN ⤍ Ch25h, 25‐HC ⤍ suppression of SREBP2 and cholesterol biosynthesis ⤍ AIM2 inhibition	25‐HC prevents mitochondrial dysfunction and AIM2 activation	[37]
	**SIRT1**. Deacetylation of proteins, NAD+‐dependent deacetylase and metabolic reprogramming.	SIRT1 ⤍ increased glycolysis ⤍ suppression of AIM2 expression	Contribution to pathology of cervical cancer	[38]
	**Ifi202b/p202**. The IFN‐inducible gene Ifi202b encodes the protein 202 (p202), which binds dsDNA in the cytosol and inhibits caspase activation and AIM2 inflammasome.	Active AIM2 ⤍ suppressed Ifi202b/p202‐prevention of high fasting glucose levels ⤍ adipogenesis ⤍ monocyte infiltration and inflammation	Protective effect against obesity and insulin resistance	[39]
	**High glucose, ROS production**	High glucose, ROS production ⤍ AIM2 activation, GSDMD signalling ⤍ cardiac dysfunction	Contribution to pathology of diabetic cardiomyopathy	[40]
Caspase 11 inflammasome	**L‐adrenaline‐induced cAMP.** Produced from ATP by adenylate cyclase, key second messenger, anti‐inflammatory effect in several immune cells.	L‐adrenaline ⤍ ADCY4‐cAMP‐PKA activation ⤍ inhibition of caspase 11 inflammasome and pyroptosis	Protective effect of L‐adrenaline against sepsis	[41]
NLRC3	TRAF6, NF‐κB signalling. TRAF6 is important for TNFR signalling and is a critical factor for the IL‐1R/TLR family.	NLRC3 activation⤍ ubiquitination of TRAF6 ⤍ NF‐κB inhibition ⤍ diminished glycolysis and oxidative phosphorylation of CD4^+^ T cells.	Protective effect against MS	[48]
	**Lactate dehydrogenase**. Glycolytic enzyme that catalyses the reversible conversion of pyruvate and NADH to lactate and NAD+.	NLRX1 deletion⤍ increased lactate dehydrogenase⤍ increased proliferation and differentiation into inflammatory phenotype in T cells.	Protective effect against colitis	[57, 59]
NLRX1	**NX‐13**. Small molecule acting as an NLRX1 agonist, induces immunometabolic changes leading to decreased inflammation and has a protective role in IBD.	NX‐13‐NLRX1 activation ⤍ increased OXPHOS ⤍ decreased Th1 and Th17 differentiation ⤍ decreased ROS	Protective effect against IBD	[59]
	**Glutamate**. In astrocytes, glutamate either is converted to glutamine or metabolized in the TCA cycle.	NLRX1 activation ⤍ increased astrocytic glutamate uptake ⤍ inhibition of Ca^2+^‐mediated glutamate exocytosis from astrocytes	Protective effect against CNS inflammation	[65]

### NLRP1

A wide range of studies has identified the NLRP1 inflammasome as a sensor of metabolic stress, leading to IL‐18 production. Human NLRP1 gets activated by double‐stranded RNA (dsRNA) and enteroviral 3C protease [22, 23], whereas NLRP1B, the murine paralog of NLRP1 that forms an inflammasome, is mainly activated in response to anthrax lethal toxin [24, 25]. NLRP1 has a protective or worsening effect in different immunometabolic diseases [26, 27] and can act as a sensor of cellular stress, glucose and lipid metabolism. In mice deficient in NLRP1B, changes in medium‐chain fatty acid (MCFA) and short‐chain fatty acid (SCFA) metabolism have been associated with microbiome dysbiosis and inflammatory bowel disease (IBD) [28]. In addition, the ω‐3 FA DHA has been shown to inhibit anthrax lethal toxin‐induced NLRP1B via β‐arrestin [29], clearly indicating the modulatory role of lipid metabolism in NLRP1B and its protective role in maintaining gut homeostasis.

NLRP1B can also act as a sensor of cellular energy stress, being activated by the glycolysis inhibitor 2‐deoxyglucose (2‐DG) and the electron transport chain inhibitor sodium azide via depletion of cytosolic ATP. Glucose‐free media and hypoxia, which lower ATP, both activated NLRP1B inflammasome transfected in human fibroblasts, and NLRP1B was suggested as a direct sensor of ATP levels [30, 31]. In addition, NLRP1 is upregulated during endoplasmic reticulum (ER) stress through PERK and IRE1α, as well as ATF4 transcription factor, which is involved in the general integrated stress response (ISR) [32].

Interestingly, NLRP1 has been shown to have a protective role in diabetes. Diabetic kidney disease (DKD) T1D patients with rs2670660 and rs11651270 NLRP1 polymorphisms (gain‐of‐function variants) were associated with having a decreased risk of developing DKD. Supporting these findings, diabetes‐associated metabolites (high glucose and glycated albumin) were used to mimic the diabetic milieu in monocytes from healthy individuals and were found to increase NLRP1 expression [26]. In addition, the same polymorphisms have recently been associated with lower susceptibility of T1D [33]. The molecular mechanisms by which NLRP1 regulates T1D, however, are still unclear and could potentially uncover new therapeutic targets for this disease.

Several studies have involved NLRP1 having either a protective or worsening role in a variety of metabolic diseases. However, the molecular and metabolic mechanisms of NLRP1 that regulate these diseases are still unclear. IL‐18 production by NLRP1B has shown a protective effect in obesity and metabolic syndrome in mice [34]. Conversely, NLRP1 is known to contribute to pathology in metastatic melanoma [35] and diabetic retinopathy, where excessive inflammation and high glucose levels seem to promote disease [27]. Overall, it is clear that NLRP1 inflammasome has a crucial role as a sensor of cellular stress, as well as glucose and lipid metabolic perturbation, maintaining cellular homeostasis to prevent a wide range of metabolic diseases. However, NLRP1 can also lead to uncontrolled inflammation contributing to disease; therefore, further investigation into the mechanisms that control the level of NLRP1 activation is needed.

### AIM2

The AIM2 inflammasome can be upstream or downstream to metabolic regulation, mainly by metabolites and enzymes from glucose metabolism. Pyruvate kinase isoform M2 (PKM2)‐mediated glycolysis, measured by phosphoenolpyruvate (PEP) and lactate levels, was shown to activate pyruvate dehydrogenase kinase 1 (PDK1), which inhibits the entry of pyruvate to the TCA cycle, leading to phosphorylation of eukaryotic translation initiation factor 2 alpha kinase 2 (EIF2AK2) and subsequent enhanced activation of mouse and human AIM2 inflammasome by poly(dA:dT). AIM2 inflammasome contributed to the release of IL‐1β, IL‐18 and septic death in mice, which was protected by conditional knockout of PKM2, suggesting a novel mechanism of AIM2 regulation by glycolytic metabolism and a potential therapeutic strategy to treat sepsis [36].

Furthermore, elevated cholesterol in macrophages is known to impair mitochondrial metabolism and activate murine AIM2 inflammasome via mtDNA as a possible ligand. Production of the soluble cholesterol‐25‐hydroxylase (Ch25 h)‐induced 25‐HC (Ch25h produces 25‐HC from cholesterol) by macrophages was reported to inhibit AIM2 inflammasome via suppression of sterol regulatory element‐binding protein (SREBP2) activation and cholesterol biosynthesis, resulting in mitochondrial integrity and homeostasis [37].

AIM2 regulation has directly been involved with certain autoimmune diseases, mostly mediated by signalling events linked to glucose metabolism. A study using sirtuin 1 (SIRT1) knockdown showed that SIRT1 has been shown to induce metabolic reprogramming by increasing mitochondrial respiration and repress the NF‐κB‐driven transcription of AIM2, contributing to cervical cancer pathology in patients [38]. In addition, deletion of AIM2 in mice results in elevated levels of fasting glucose and insulin, leading to white adipose tissue inflammation and obesity via the IFN activated gene Ifi202b [39]. In contrast, AIM2 activation by high glucose in rats has recently been shown to contribute to pathology in diabetic cardiomyopathy via ROS production and the GSDMD pathway [40]. Overall, increased glycolysis and disruption in TCA cycle and mitochondrial dysfunction have generally been implicated in the activation of AIM2 inflammasome across different studies. It would be interesting to determine whether 25‐HC modulation of this inflammasome is involved in diseases where lipid metabolism is disrupted, as well as the involvement of other lipid metabolites. In addition, further research considering the SIRT1‐AIM2 axis as a target for cervical cancer will be crucial for the development of new therapies.

### Non‐canonical caspase 11 inflammasome

A recent mouse study identified a mechanism of metabolic regulation of the caspase 11 inflammasome by regulation of cAMP, which promotes PKA leading to caspase 11 inflammasome inhibition. L‐adrenaline‐induced cAMP via the enzyme ADCY4 showed inhibition of cytosolic LPS‐induced caspase 11 inflammasome pathway and consequent pyroptosis in macrophages, and presented this novel interaction with caspase 11 inflammasome as a potential therapeutic target in sepsis [41]. Interestingly, the cAMP‐PKA axis leading to inhibition of inflammasome and pyroptosis has also been described in several studies involving the canonical NLRP3 inflammasome [42, 43], given its contributing role in chronic inflammatory diseases, it would be interesting to explore further the cAMP regulation of this inflammasome in sepsis.

### NLRC3

NLRC3 whose functions are mostly undiscovered as compared to other NLRs, has been identified as a negative regulator of innate immunity and inflammatory responses [44, 45]. NLRC3 does not form an inflammasome complex and was shown to inhibit LPS‐induced Toll‐like receptor (TLR) signalling by inhibiting the adaptor protein TNF receptor‐associated factor 6 (TRAF6) [46]. Furthermore, NLRC3 blocks activation of the PI3K‐dependent Akt kinase and inhibits mTOR pathways in colon epithelial cells [45].

Interestingly, NLRC3 plays a major role in T‐cell metabolism. Aerobic glycolysis is a metabolic hallmark of activated T cells and Th1 cell differentiation [47]. NLRC3 acts as a brake, diminishing metabolic pathways to attenuate T‐cell activation in a NF‐κB‐dependent manner. NLRC3 deficiency in mice increases oxidative phosphorylation and glycolytic capacity, driving Th1 and Th17 cell differentiation, proliferation and IFN‐γ, IL‐2, TNF and IL‐17 production, thus leading to unrestrained generation of activated CD4^+^ T cells with maximal glycolytic and mitochondrial metabolism [48], which could lead to autoimmune disease progression. Profiling of T cells from patients with multiple sclerosis (MS) has reported increased glycolysis and mitochondrial activity [49]. Therefore, it could be possible that a deficiency in NLRC3 expression within the human population could predispose individuals with low CD4^+^ T‐cell NLRC3 expression to develop multiple sclerosis (MS) or MS‐related disorders.

### NLRX1

NLRX1, an enigmatic modulator of immune function, is part of an NLR family subgroup which are non‐inflammasome` forming and regulate the inflammation associated with other PRRs activation. The location and trafficking of NLRX1 is debated but it is known to negatively regulate type I interferon, IL‐6, NF‐κB signalling and to induce ROS production, activating the JNK pathway leading to apoptosis [50, 51, 52, 53, 54].

Loss of function of NLRX1 has been linked with a variety of autoimmune diseases, such as cancer, colitis, inflammatory bowel disease and CNS inflammation [55]. In human breast cancer cells, NLRX1 expression is increased and, in the presence of TNF, helps maintenance of mitochondrial metabolism‐lysosomal crosstalk to regulate invasion and metastasis capability. NLRX1 deletion results in lysosomal dysfunction and decreased turnover of damaged mitochondria via mitophagy, leading to a reduction of OXPHOS‐mediated cell proliferation and migration ability [56]. In mouse T cells, loss of NLRX1 mediated increased lactate dehydrogenase activity, suggesting a preference for aerobic glycolysis, and a greater ability to proliferate and differentiate into a pro‐inflammatory phenotype, combined with a reduced sensitivity to immune checkpoint pathways. Interestingly, in the same study NLRX1 showed a protective effect against colitis, where *rag2* ^−/−^ mice (an adoptive‐transfer model of colitis) receiving *nlrx* *1* ^−/−^ naive or effector T cells, showed increased disease activity [57]. Another study showed that NLRX1 deletion in colitis mice drives changes in gut–microbiome interactions exacerbating disease; however, restoration of WT glutamine metabolic profiles via glutamine supplementation abrogated effects in microbiome, inflammation and disease severity [58]. In addition, a novel orally active, gut‐restricted drug named NX‐13 has been shown to activate mouse NLRX1 and alleviate disease severity in IBD mouse models, as well as increased OXPHOS, decreased differentiation into Th1 and Th17 subsets and decreased ROS and NF‐κB activation in naïve CD4^+^ T cells. In addition, NX‐13 activation of NLRX1 also showed resistance to oxidative stress and inflammation in human primary cells from ulcerative colitis patients, therefore becoming a promising NLRX1 agonist for treating IBD [59, 60, 61].

Finally, NLRX1 has been identified as a regulator of glutamate homeostasis in the CNS. Astrocyte dysfunction leading to accumulation of extracellular glutamate and lack of NLRX1 is known to be associated with CNS trauma and excessive inflammation [62, 63, 64]. Using NLRX1‐depleted mice, NLRX1 was reported to potentiate mitochondrial function causing increased astrocytic glutamate uptake, as well as inhibition of Ca^2+^‐mediated glutamate exocytosis, leading to repression of glutamate release from astrocytes, suggesting a protective role of NLRX1 in CNS inflammation [65]. Overall, NLRX1 has been described to act as a brake in inflammation and negative regulator of pathology, playing a key role in the regulation of immunometabolism with links to disease. The emergence of NLRX1 agonists such as NX‐13 show the potential in targeting NLRX1 to modulate immunometabolic function in diease, and future research focusing on its involvement in colitis and gut microbiota is needed.

## CROSSTALK BETWEEN METABOLISM–INFLAMMASOME INTERACTIONS AND OTHER IMMUNE PATHWAYS

### GPCR signalling

G protein‐coupled receptors (GPCRs) are an extensive family of membrane receptors known to share seven‐transmembrane α‐helix domains, with an intracellular C terminus and a large extracellular N terminus. Generally, the extracellular region senses extracellular ligands such as ions, metabolites, chemokines and temperature, causing conformational changes in the receptor and subsequent transduction of the signal into intracellular responses, activating further downstream signalling pathways. Canonical GPCRs are classically modulated by the intracellular region ligands: G proteins, which activate downstream signalling, or β‐arrestins, which can control excessive activation [66, 67]. GPCRs are the most common class of targets for therapeutic drugs (~35% of approved drugs) due to their relevance in several human diseases, restricted expression and structure. However, only about 16% of the ~800 different GPCRs have been targeted for therapeutic drugs, leaving a substantial number of distinct GPCRs to be explored as potential therapeutic targets [68, 69].

**FIGURE 2:**
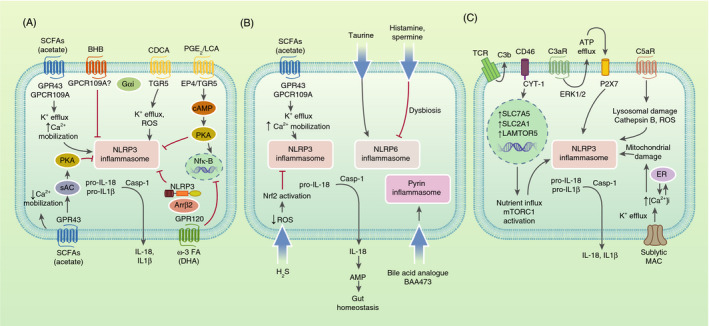
Metabolic regulators of the inflammasome in the crossroads with: GPCR signalling, host–microbiota communication and the complement system. (A) GPCRs that activate or inhibit NLRP3 inflammasome by metabolites. GPR43 and/or GPR109A act as SCFA (mainly acetate) receptors to induce NLRP3 inflammasome activation via K^+^ efflux, increased Ca^2+^ mobilization and membrane hyperpolarization in gut epithelial cells, or to repress NLRP3 inflammasome activation via decreased Ca^2+^ mobilization and activation of sAC and PKA in BMDMs. GPR120, receptor of the ω‐3 FA DHA, inhibits NLRP3 activation through suppression of NF‐κB and binding of β‐arrestin 2 (Arrβ2) to NLRP3, impeding its assembly. BHB inhibits NLRP3 activation via an undefined Gαi‐coupled GPCR. TGR5 and EP4, receptors of bile acids (LCA) and PGE_2_, respectively, both inhibit NLRP3 via cAMP‐PKA signalling, causing NLRP3 phosphorylation. PGE_2_ can also promote NLRP3 priming via EP4‐cAMP‐PKA signalling leading to NF‐κB activation in BMDMs. The bile acid CDCA can bind TRG5 leading to NLRP3 activation through K^+^ efflux and ROS production. (B) Inflammasome activation in the gut by metabolites leads to IL‐18 release which promotes AMP production and secretion by nearby cells, controlling the gut microbial composition. Upon dysbiosis induction, histamine and spermine inhibit NLRP6‐dependent IL‐18 and AMP, supporting the dysbiotic microbiota. Taurine reduces spermine and histamine levels and induces IL‐18 secretion and improved microbiota composition. H_2_S inhibits NLRP3 inflammasome and IL‐1β secretion in DSS‐induced colitis colons via reduced ROS and Nrf2 activation.The bile acid analogue BAA473 activates pyrin inflammasome in intestinal epithelial cells leading to IL‐18 secretion. (C) The complement system activates NLRP3 inflammasome via metabolic changes. Activation of C3aR drives ATP efflux from the cytosol and ERK1/2 phosphorylation. ATP efflux triggered by C3a activates P2X7, leading to NLRP3 activation. C5a binding to C5aR1 triggers NLRP3 inflammasome priming and activation via ROS production, lysosomal damage and cathepsin B activity. CD46‐CYT‐1 upregulated by TCR stimulation through autocrine C3b production drives upregulation of LAT1 and GLUT1, which mediate nutrient influx into the cell, as well as LAMTOR5. LAMTOR5 drives mTORC1 activation (a known NLRP3 inflammasome activator) leading to increased glycolysis and Th1 cell induction. CD46‐CYT‐1 signalling also primes NLRP3 inflammasome by upregulating IL‐1β and NLRP3. Membrane attack complex deposition drives NLRP3 activation in human lung epithelial cells via Ca^2+^ influx, increased [Ca^2+^]i in the cytosol and endoplasmic reticulum (ER) stores, subsequent mitochondrial [Ca^2+^]i uptake and alteration of mitochondrial membrane potential

Activation of inflammasomes, mainly NLRP3, can be modulated via GPCRs interacting with different metabolites, ions, neurotransmitters and hormones, indicating crosstalk between metabolism, GPCR signalling and inflammasomes. Metabolites and metabolic proteins involved in such process include ω‐3 FAs, cyclic adenosine monophosphate (cAMP), β‐hydroxybutyrate (BHB), bile acids, prostaglandin E_2_ (PGE_2_) and SCFAs such as acetate (Figure 2).

SCFAs are derived from commensal anaerobic bacterial fermentation of dietary fibre and are known to play a crucial role in gut homeostasis and inflammation. SCFA acetate binding to metabolite‐sensing receptors GPR43 and/or GPCR109A has been shown to mediate a protective effect against colitis and drive activation of NLRP3 inflammasome in epithelial cells, showing caspase 1 activation and secretion of IL‐18. Acetate led to GPR43‐dependent inflammasome activation through K^+^ efflux, Ca^2^^+^ mobilization and downstream hyperpolarization [70]. Conversely, acetate interacting with GPR43 in bone marrow‐derived macrophages (BMDMs) has recently been described as a suppressor of NLRP3 inflammasome via a Ca^2^^+^‐dependent mechanism, leading to decreased Ca^2^^+^ mobilization. The mechanism also involved adenylyl cyclase (sAC)‐PKA signalling leading to NLRP3 ubiquitination and autophagy. In addition, the study showed that acetate had a protective effect on NLRP3‐dependent peritonitis in vivo [71].

Certain GPCRs can have a dual effect on regulating the inflammasome in different situations and inflammatory states; therefore, further research is crucial to better understand their mechanisms and establish more GPCRs as new therapeutic targets. Another example of that is the PGE_2_ receptor 4 (EP4) and the bile acids active membrane receptor TGR5, which are known to inhibit or activate NLRP3. EP4 binding to PGE_2_ can inhibit or activate priming of NLRP3 via cAMP increase and subsequent PKA activation: in HEK cells, cAMP leads to phosphorylation of human NLRP3 on Ser295 and PKA decreases its ATPase function causing NLRP3 inhibition, whereas in BMDMs, PGE_2_ promotes NLRP3 priming through the EP4‐cAMP‐PKA mechanism leading to NF‐κB activation [43, 72]. Interestingly, TGR5 binding to lithocholic acid (LCA) can suppress NLRP3 in a similar mechanism which is also cAMP‐PKA dependent, where PKA leads to NLRP3 phosphorylation on mouse Ser291 (corresponding to human S295), causing ubiquitination of NLRP3. The same study showed prevention of high‐fat diet‐induced insulin resistance downstream of TGR5‐NLRP3 inhibition axis [42]. Conversely, TGR5 binding to the major bile acid involved in cholestatic liver injury, chenodeoxycholic acid (CDCA), leads to activation of NLRP3 by an independent cAMP‐PKA mechanism, where ROS production, K^+^ efflux and c‐Jun N‐terminal kinase pathways have been involved [73]. It is important to note that CDCA binding to TGR5 has been reported to both activate and inhibit NLRP3‐mediated caspase 1 activation and IL‐1β secretion in BMDMs through different mechanisms, the biological basis of these discrepancies has not been elucidated and therefore more research is needed to clarify the CDCA‐TGR5‐inflammasome axis [42, 73].

Metabolites that have been reported to only inhibit the inflammasome via GPCRs are ω‐3 FAs and β‐hydroxybutyrate. The ω‐3 FA docosahexaenoic acid (DHA) interacting with its receptor GPR120 was shown to inhibit inflammasome priming and activation, first through suppression of NF‐κB and then inhibiting IL‐1β release via increased autophagy. Interestingly, not only NLRP3 but also NAIP5/NLRC4 and AIM2 inflammasomes were involved in this process. In addition, DHA mediated β‐arrestin 2 recruitment to GPR120, leading to a β‐arrestin 2‐dependent reduction of inflammasome activation [74]. Finally, while the ketone body BHB was found to inhibit NLRP3 independently of GPCRs in macrophages and neutrophils, it has recently been reported that in C6 glioma cells, BHB can inhibit NLRP3 through a Gαi‐coupled GPCR, whether that is the BHB Gαi‐coupled receptor GPR109A or not remains to be explored [75].

It is clear that certain metabolism–inflammasome interactions with GPCR signalling are still controversial, this is especially apparent in the case of SCFAs having opposite roles as modulators of NLRP3 inflammasome in colitis and peritonitis, which could be attributed to the fact that studies were performed in different cell types and experimental conditions. Thus, further research should focus on the upstream NLRP3 activation mechanisms in disease, as well as understanding how GPCR‐driven cAMP production inhibits NLRP3, or whether other inflammasomes other than NLRP3 are involved in GPCR signalling.

### Host–Microbiota communication

The gut microbiota is known to communicate with the host having a commensal interaction, where microbes and their derived metabolites resident in the gut are able to sense changes in the microbiota and act as signals to regulate a variety of signalling pathways including host PRRs, such as inflammasomes. The inflammasomes regulated by microbiota‐associated metabolites have mainly been NLRP6, NLRP3 and pyrin (Figure 2). Inflammasome activation at a basal level and production of IL‐18 in the gut are crucial to maintain homeostasis of the intestinal microbiome; however, dysregulation of inflammasome activation caused by certain host and gut‐derived metabolites can lead to dysbiosis [76, 77]. Dysregulation of the microbiota homeostasis can alter the intestinal barrier's function with unwanted leakage of pro‐inflammatory signals, as well as alteration of many metabolic products contributing to dysbiosis‐associated intestinal carcinogenesis and non‐alcoholic fatty acid disease (NAFLD) [78, 79].

Given the high expression of NLRP6 in the intestine, it is not unexpected that its ligands are mostly derived from bacterial components in the gut. Expression of NLRP6 in gut epithelial cells has been shown to regulate the intestinal microbiota and mucus secretion [80]. Furthermore, a metabolomics–metagenomics analysis of caecal content identified microbiota‐modulated metabolites as co‐modulators of NLRP6 inflammasome activation, with downstream epithelial IL‐18‐induced antimicrobial peptide (AMP) production, driving stability of the intestinal microbial community. In vitro and in vivo mice studies showed that upon dysbiosis induction, the metabolites histamine and spermine inhibited NLRP6 inflammasome‐dependent IL‐18 production, supporting the dysbiotic microbiota. Administration of taurine showed reduced spermine and histamine levels and induced IL‐18 production, improved microbiota composition and had a protective effect against colitis via NLRP6 inflammasome [76]. Interestingly, a recent study showed improvement of gut permeability and reduction of intestinal inflammation after reactivating NLRP6 through Roux‐en‐Y gastric bypass (RYGB) surgery in obese rats, housed in a specific pathogen‐free facility. After RYGB, increased taurine positively affected NLRP6 expression [81].

In contrast, studies conducted in germ‐free facilities contradicted the aforementioned studies and observed no impact of NLRP6 in gut microbiota composition, attributing this result to the fact that they used littermate‐controlled WT and NLRP6‐deficient mice to minimize non‐genetic confounders, allowing them to shape their gut microbiota naturally after birth. Such studies alleged that the results obtained by previous studies showing the opposite effect were not obtained from littermate animals; therefore, it was possible that the dysbiosis observed in the NLRP6‐deficient mice was genotype independent and acquired stochastically [82, 83].

A recent study identified a secondary metabolite of flavonoids and a common constituent of the human diet, apigenin, as a regulator of gut microbiota via NLPR6. Apigenin showed a protective effect against colitis in mice via NLRP6 signalling, which still happened in the absence of caspase 1/11 or Asc, suggesting that an alternative mechanism to the classical inflammasome pathways was involved. Mice showed protection against colitis when cohoused with apigenin‐treated mice, whereas NLRP6‐deficient mice lost the protective effect [84]. Thus, the role of NLRP6 inflammasome as a regulator of intestinal microbiota is still controversial and further research, possibly in human studies, is needed to confirm this role.

Activation of NLRP3 inflammasome in the colon has been shown to have both protective and detrimental effects in different colitis studies. NLRP3 deficiencies showed either defects in intestinal mucosa repairing, exacerbating disease, or decreased levels of secreted IL‐1β and autoinflammation, reducing disease severity [70, 85, 86, 87]. In an aforementioned study, bacterial SCFAs metabolites showed steady‐state NLRP3 inflammasome activation and IL‐18 production in epithelial cells, leading to regulation of the microbiome composition, reduction of inflammatory responses and a protective effect against colitis [70]. Conversely, the bacterial metabolite hydrogen sulphide (H_2_S) has been shown to inhibit NLRP3 inflammasome and IL‐1β secretion in BMDMs and in dextran sulphate sodium (DSS)‐induced colitis colons via reduced ROS and Nrf2 activation. In addition, H_2_S attenuated the severity of mouse colitis by NLRP3 inflammasome inhibition [88]. Different levels of tissue injury and inflammation caused by intestinal NLRP3 can determine its protective or detrimental function in the gut, which can also depend on the different experimental conditions between studies [89].

Interestingly, a screen for microbiome metabolites that may function as inflammasome activators recently identified BAA473, a bile acid analogue, as a pyrin inflammasome activator leading to IL‐18 secretion in human intestinal epithelial and myeloid cells. Given the crucial role of IL‐18 in the gut, the study suggests that microbiota‐mediated bile acid conversion might modulate intestinal homeostasis via the pyrin inflammasome pathway [90].

Finally, NLRP12 has been reported to have a protective role in IBD patients and act as a regulator of the colonic microbiota and suppressor of inflammation to microbial ligands. NLRP12 deficiencies in mice lead to IL‐6 and TNF‐driven colonic inflammation and dysbiosis via an inflammasome‐independent pathway [91]. However, whether there are any bacterial metabolites that regulate NLRP12 and its functions in the gut remain to be explored. Overall, the controversial evidence regarding the involvement of NLRP6 and its microbiota‐derived metabolites as regulators of gut homeostasis, as well as the different roles of NLRP3 against colitis, which possibly lays in different cell types and grades of inflammasome activation, indicates the need for further research in human disease settings and into the downstream mechanisms that control these effects.

### The complement system

In recent decades, it has become apparent that the complement system and inflammasomes are not only pathogen sensors, but also systems that can recognize cell metabolic changes and induce reactive responses, for instance, to support cell activation or to maintain cell homeostasis. Several complement receptors and regulators have been presented as critical signals for NLRP3 inflammasome activation, either linked with signals coming from RLR or TLR activation, or independently [92]. This process can occur through metabolic changes being upstream or downstream to complement, leading to inflammasome activation (Figure 2).

Activation of the complement anaphylatoxin receptor C3aR in myeloid cells results in NLRP3 inflammasome activation. This process has been reported to occur in the presence of LPS and TLR4 activation through increased ATP efflux from the cytosol and ERK1/2 phosphorylation. ATP efflux triggered by C3a resulted in P2X purinoceptor 7 (P2X7) activation, leading to inflammasome activation [89]. Interestingly, an ATP‐inflammasome‐complement axis has recently been proposed, where complement is activated by DAMPs released by inflammasome activation, promoting sterile brain inflammation [93].

C5a generation driven by cholesterol and acid uric crystals has also been shown to act as a trigger for the NLRP3 inflammasome. Abnormally high concentrations of the metabolite cholesterol, which can be driven by defects in cholesterol metabolism and homeostasis, precipitate to form cholesterol crystals (CC) driving inflammation and contributing to pathogenesis in atherosclerosis [94]. A study showed that CC activate complement and that the resulting C5a generated, combined with TNF‐α, triggered ROS and priming of the inflammasome [95], suggesting complement inhibition as a potential therapeutic target for atherosclerosis treatment. Furthermore, studies in which inflammation was induced by the crystalized metabolite uric acid in monocytes as a model of gout disease have shown that C5aR1 triggers priming and activation of the inflammasome via lysosomal damage and cathepsin B activity [96]. This role for C5a was confirmed by other studies using neutrophils in a mouse peritonitis model [97].

Complement can not only regulate metabolic changes at a systemic level [98], but also at an intracellular level. Autocrine complement activity has been shown to induce metabolic reprogramming leading to T‐cell activation and inflammasome activation [99, 100]. Human CD4^+^ T cells express CD46 in two different isoforms based on their cytoplasmic tails, known as CYT‐1 and CYT‐2. CD46‐CYT‐1 is upregulated by T‐cell receptor (TCR) stimulation via autocrine C3b production, leading to increased expression of the amino acid and glucose transporters LAT1 and GLUT1, as well as MAPK and MTOR activator 5 (LAMTOR5), leading to Th1 cell induction. LAT1 and GLUT1 mediate nutrient influx, while LAMTOR5 drives mTORC1 activation and glycolysis, a metabolic hallmark in activated T cells and known NLRP3 inflammasome activator [92]. Alternatively, CD46‐CYT‐2 is expressed in resting and contracting T‐cells, where CD46‐CYT‐2 mediates a switch from glycolysis to OXPHOS metabolism [99]. Interestingly, CD46 (which binds C3b) stimulation in activated CD4^+^T cells was found to simultaneously upregulate IL‐1β and NLRP3 gene expression to prime the NLRP3 inflammasome, as well as increase intracellular generation of C5a and activation of C5aR1, which led to increased ROS production and subsequent NLRP3 inflammasome activation and Th1 cell induction [100]. Dysregulation of C3 and CD46 in CD4+ T cells has been reported to contribute to Th1‐mediated autoimmune diseases [101, 102, 103]. Furthermore, cytotoxic CD8+ T cells have recently been involved in the CD46 regulator costimulation signalling, leading to increased fatty acid synthesis and nutrient influx which promote optimal cell activity. However, this process was not linked with the canonical NLRP3 signalling [104].

Finally, studies in dendritic cells, C6‐deficient mice [105] or in human lung epithelial cells have elucidated that sublytic membrane attack complex (MAC) activates NLRP3 inflammasome via Ca^2+^ influx, subsequent mitochondrial [Ca^2+^]i uptake and alteration of mitochondrial membrane potential [106]. Interestingly, sublytic MAC and increased glycolysis have been implicated in rheumatoid arthritis, contributing to inflammation [107, 108]. Given that MAC is an inflammatory trigger and such stimuli have been implicated in the modulation of immunometabolic responses, perturbations in cellular metabolism downstream of sublytic MAC might be involved in this process. In conclusion, a functional complement–metabolism–inflammasome axis has been demonstrated particularly in T cells (CD46‐C3b and C5aR1‐ROS interactions). More complement components such as MAC, as well as other cell types are likely to be involved in this axis in the future, providing potential novel therapeutic targets in the treatment of autoimmune diseases.

## CONCLUSIONS AND FUTURE PERSPECTIVES

Growing evidence on modulation of inflammasome activation by metabolic changes has become apparent in the recent years not only for NLRP3, but also for NLRC4, NLRP1, AIM2, NLRP6, NLRP12, pyrin, NLRC3, NLRX1 and non‐canonical caspase 11 inflammasome. Metabolic control of these inflammasomes and non‐inflammasome forming NLRs implicates a variety of perturbations in the glycolytic pathway, mitochondrial and lipid metabolism, which has been linked to several autoimmune and metabolic diseases, such as obesity, diabetes and cancer. The downstream mechanisms of the metabolic perturbations that directly regulate the inflammasome, however, remain unclear, where some independent mechanisms have been proposed but not always linked to each other. Whether such mechanisms interact with each other in a common signalling cascade or distinct pathways needs to be understood. Furthermore, novel links between inflammasomes and metabolic diseases are recently emerging, such as NLRP1 and NLRC4’s involvement in T1D and diabetic complications, which have been linked to certain metabolic markers [21, 26]. Elucidation of the mechanism of action between the metabolic alterations and inflammasomes linked to disease will be crucial to allow development of new therapeutic targets.

There is contradictory evidence regarding certain metabolism–inflammasome interactions and whether they have a protective or worsening effect in disease. SCFAs interacting with GPCR signalling have been reported to both activate and inhibit NLRP3 through different mechanisms, leading to a protective or detrimental role in colitis/peritonitis [70, 71]. The fact that metabolites often interact with Gs‐coupled GPCR signalling to regulate the inflammasome indicates potential development of new GPCR‐targeted treatments for inflammasome‐driven diseases. However, more research is needed to understand GPCR regulation of the upstream NLRP3 activation mechanisms, as well as how GPCR‐driven cAMP production inhibits NLRP3, whether other inflammasomes other than NLRP3 are involved or how to specifically modulate inflammasome activation by targeting GPCRs.

NLRP3 and NLRP6 are the main gut inflammasomes, NLRP3 and arguably NLRP6, seem to be regulated by the metabolic outputs of gut microbiota and have a crucial involvement in DSS‐colitis. The recent finding about intestinal pyrin inflammasome being activated by bile acids presents pyrin as an emerging metabolic sensor in the gut [90]. Its upstream mechanisms, role in gut microbiota and gut‐related diseases, however, remain unclear and need to be understood. In addition, gut microbiota activating intestinal NLRP3 inflammasome is known to aggravate progression of CNS diseases such as AD and has been described as the microbiota‐gut‐brain axis [109, 110, 111, 112]. Whether certain gut metabolites (SCFA catabolism [109]) have a role in NLRP3 activation in CNS diseases should be investigated. Finally, the complement–metabolism–inflammasome axis has been well described for the NLRP3 inflammasome mainly in T cells. More research is needed to unveil whether other inflammasomes are involved in this process, as well as other primary cells and tissues. Equally important will be to better understand if other complement components are involved, i.e. further elucidation of the role of MAC complex in this axis, and their downstream metabolic mechanisms leading to inflammasome activation.

## CONFLICT OF INTEREST

The authors declare no competing financial interests.
